# Is Fasting Duration Important in Post Adenotonsillectomy Feeding Time?

**DOI:** 10.5812/aapm.10256

**Published:** 2014-02-26

**Authors:** Yalda Jabbari Moghaddam, Mahin Seyedhejazi, Mosoud NaderPour, Yoosef Yaghooblua, Samad Golzari

**Affiliations:** 1 Department of Otolaryngology, Head and Neck Surgery, Tabriz University of Medical Sciences, Tabriz, Iran; 2 Department of Anesthesiology, Tabriz University of Medical Sciences, Tabriz, Iran

**Keywords:** Pain, Nausea, Vomiting, Children, Fasting, Preoperative Period

## Abstract

**Background:**

Adenotonsillectomy is a common otolaryngology surgery. Nausea and vomiting are the most common complications of this procedure with a prevalence ranging from 49% to 73 %.

**Objectives:**

Our aim was to evaluate the effects of short time fasting protocol on decreasing postoperative pain, nausea and vomiting, and initiation of oral feeding after adenotonsillectomy.

**Patients and Methods:**

120 children aged 4 to 14 years candidates for adenotonsillectomy were randomly divided into intervention and control groups (n = 120, 60 in each group). Each patient of the intervention group was given oral dextrose 10% as much volume as he could consume at 3 and 6 hours prior to the operation. All the data including pain severity, nausea and vomiting of the patients, the time of oral feeding initiation etc. were gathered in checklists after the operation. Statistical analyses were then performed using Statistical Package for the Social Sciences (SPSS) software version 16. Descriptive statistical methods and mean difference test for independent groups and chi square test or Fisher exact test, and if regression needed model test were applied. A P value of 0.05 or less was considered statistically significant.

**Results:**

The amount of Acetaminophen administered for the intervention group was significantly lower than the control group, and also the time of oral feeding initiation was significantly shorter in the intervention group than the control group (P < 0.005). Pain severity at all occasions following surgery was significantly lower in the intervention group than the control group (P < 0.001). Although frequency of nausea at recovery time was significantly lower in the intervention group than the control group (P < 0.002), there were no significant differences in frequency of nausea between the two groups at other postoperative occasions. Postoperative vomiting frequency was not significant between the two groups at any occasions.

**Conclusions:**

The findings of this survey showed that shortening the duration of pre-adenotonsillectomy fasting period and hydration of patients several hours prior to the operation might be effective in decreasing postoperative pain and facilitating postoperative oral feeding initiation. Nevertheless this method does not seem to prevent postoperative nausea and vomiting.

## 1. Background

Adenotonsillectomy is a common otolaryngology surgery, with nausea and vomiting as the most prevalent complications of this procedure with a prevalence ranging from 49% to 73% (1-4). This complication affects more than 41% of patients (5-7). Thus pain, nausea and vomiting are common problems in children after adenotonsillectomy, and more than a half of children who undergo surgery have vomiting (8, 9).

Although effects of applying some systemic medications prior to, during or after adenotonsillectomy (10), local anesthesia (11), administering some antibiotics (12), new surgery techniques (13), and pain relieving techniques have been studied in pain control, nausea and vomiting postoperatively, but there is yet no satisfactory cure for postoperative pain, nausea and vomiting.

Unfortunately postoperative pain, nausea and vomiting delay oral feeding initiation which consequently induce dehydration and delay in operation site healing, and on the other hand cause prolongation of convalescence period (14). It seems that other nonpharmacologic factors play role in inducing postoperative nausea and vomiting. To reduce the risk of gastric content aspiration during general anesthesia, each child should become fasting prior to transportation to the operating room (15).

However studies have shown that postoperative nausea associate with prolonged fasting (16) and some other studies have suggested that short period of fasting is safe for children and even allows children intake clear fluids and solid food two and four to six hours prior to operation respectively (17, 18). Egeli et al. (19) in their study suggested that 24 hours hydration can reduce postoperative morbidity following tonsillectomy in children. Even recently Elqueta et al. (20) studied the effect of large intraoperative crystalloid administration as prophylaxis of postoperative vomiting in children undergoing tonsillectomy in a randomized controlled trial, and concluded that super-hydration during tonsillectomy is an alternative way to decrease the risk of postoperative vomiting in children (20). Despite this change in fasting period, most of the children candidates for surgery have yet to withstand long periods of hunger.

Sounds like if we could simply take some non-pharmacological steps to keep child vivacious postoperatively e.g. by administering fasting protocol exquisitely, it would be very helpful in decreasing postoperative complications.

## 2. Objectives

The aim of this single blinded clinical trial was to score short time fasting protocol effects on decreasing pain, nausea and vomiting, and establishing oral feeding after adenotonsillectomy in hospitalized children of Tabriz Children Hospital.

## 3. Patients and Methods

After getting approval from the Medical Ethics Committee, and obtaining written informed consent from the parents of the participants, 120 children aged 4 to 14 candidates for adenotonsillectomy were randomly divided into intervention and control groups (n = 120, 60 in each group). To distribute the trial blemishing variables such as age, gender, educational status and cultural class, patients were randomly allocated into intervention and control groups. Exclusion criteria were diabetes, gastrointestinal tract disorders, upper respiratory tract infection, and weighting more than 50 kg.

In the intervened group, all of the patients were given oral dextrose 10% solution for a maximum dose of 10 mg/kg depending on their appetite. There was no obligation to intake an amount of maximal dose. Case group members were kept fasting prior to adenotonsillectomy as otolaryngology department routine (all children must be NPO from 12:00 am preoperative day, till the operation, and during this time only receiving 1/3-2/3 serum as much to keep vein open (KVO)).

As a medical ethics rule, study method was completely explained to the parents and their questions were answered. All of the parents provided their written consent form to enroll their children in the study.

**Figure 1.**
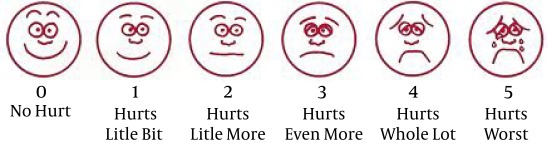
Wong-Baker FACES Pain Rating Scale

Wong-Baker FACES pain rating scale, a 0 to 10 scale, was employed to score the pain severity of patients (Figure 1) (21, 22). Another scale which its criteria are among the accredited ones in assessing postoperative nausea and vomiting was applied to assess the nausea and vomiting. 

### 3.1. Four Point Severity Scale

The four-point severity scale included no symptom, mild symptom, severe nausea or up to two vomits, and more than two vomits (23). Adenotonsillectomy was performed by a same surgeon, and invariable anesthetic drugs were used in the both groups. In all patients, premedication was performed using Midazolam (0.03 mg/kg/IV) and Fentanyl 1 μg/kg/IV few minutes before separation from parents, and anesthesia induction was initiated using Lidocaine 1 mg/kg and Propofol with a dose of 3.5 mg/kg, Atracurium 0.5 mg/kg and 0.1 mg/kg Dexamethasone single dose, and a stat 40 mg/kg of Acetaminophen suppository just after intubation (with an appropriate size ETT), and maintenance was performed with Isoflurane 1-1.5%, and a mixture of N_2_O 50% and O_2_ 50%. Fifteen mg/kg of oral Acetaminophen was administered postoperatively to the patients with a pain score more than 5 in the both groups.

According to our study aims, a checklist was designed according to the former studies. This checklist included questions concerning demographic data of the patients and other needed information. All data regarding the pain, nausea and vomiting complaints, oral feeding initiation instant, and oral analgesic drugs administered by an invariable accessory, were then gathered in the mentioned checklists postoperatively.

### 3.2. Statistical Analysis

Data was then imported in SPSS analysis software version 16 after gathering and later were analyzed. Acquired data was statistically analyzed using descriptive statistical methods (frequency, age (%), mean and standard deviation), independent groups T-test for means, chi square test, and Fisher’s exact test or regression models. A P value of 0.05 or less was considered statistically significant.

## 4. Results

One hundred and twenty patients aged 4 to 14 years were allocated into two groups of control and intervention each containing 60 patients. Allocating patients into two groups was performed by randomly permuted blocks method. There was no significantly meaningful difference in age distribution of the two groups (P = 0.247).

Among the intervention group, oral feeding was established in the first, second, third, and the fourth hours postoperatively for 40, 14, 4, and 2 patients respectively. Therefore oral feeding was initiated in all patients during four hours postoperatively.

But among controlled group, oral feeding initiation in the first hour was significantly less than the intervention group, i.e. 8 patients (Table 1). 

**Table 1.** Postoperative Oral Feeding Initiation Hours

Hours	Intervention Group	Control Group
**1**	40	8
**2**	14	2
**3**	4	5
**4**	2	5
**5**	-	20
**6**	-	20
**Total**	60	60

Amount of administered Acetaminophen in 30 patients was a single dose of 15 mg/kg which comprised most patients, and their fasting period was shorter than routine in our department.

There was significantly higher amounts of Acetaminophen applied in the control group compared to the intervention group, i.e. in 10, 24, and 20 patients the Acetaminophen was administered one, two, and three times respectively at a dose of 15 mg/kg (P < 0.005) (Table 2). 

**Table 2.** Acetaminophen Prescription Times

Acetaminophen Perception Times	Intervention Group, Patients No.	Control Group, Patients No.
**Once**	30	10
**Twice**	10	24
**Three times**	-	20
**Four times**	1	3
**Five times**	1	-
**Not use**	18	3
**Total**	60	60

Pain severity in the patients of both groups was assessed in 6 different occasions (at recovery time, 2, 4, 6, 8, and 24 hours postoperatively) according to the guidelines described in methods section of this article. At all occasions, pain severity in the intervention group was meaningfully less than the control group (P < 0.001).

Nausea prevalence was different in various occasions postoperatively, the most of which was seen in recovery time and 2 hours postoperatively, affecting a total number of 21 patients (17.5%).

As shown in Table 2 and Table 4, although there was a significant difference in pain severity between the two groups, no significant difference was noted regarding nausea and vomiting. 

**Table 3.** Comparison of Nausea Frequency in Various Postoperative Occasions Between the Intervention and Control Groups

	Recovery	2 Hours Post Operation	4 Hours Post Operation	6 Hours Post Operation	8 Hours Post Operation	24 Hours Post Operation
**Intervention group**	4	9	3	4	1	0
**Control group**	17	11	4	10	3	2
**P value**	0.002	0.387	0.491	0.7	0.303	0.248

**Table 4.** Comparison of Vomiting Frequency in Various Postoperative Occasions Between the Intervention and Control Groups

	Recovery	2 Hours Post Operation	4 Hours Post Operation	6 Hours Post Operation	8 Hours Post Operation	24 Hours Post Operation
**Intervention group**	3	3	2	2	0	0
**Control group**	4	7	1	5	0	1
**P value**	0.509	0.154	0.506	0.219	-	0.5

Comparing the frequency of nausea between the control and intervention groups revealed that there was a statistically significant lower nausea frequency (no symptom) only at recovery time, as shown in Table 3. In recovery time, only 4 of 60 patients in the intervention group complained of nausea (mild or severe symptoms), while 17 of 60 patients in the control group complained of nausea (mild or severe symptoms) (P = 0.002). In other 5 occasions, i.e. 2, 4, 6, 8, and 24 hours postoperatively, there was a lower nausea frequency reported in intervention group compared to control group, but this difference was not statistically significant. 

In this study, among 120 patients, the highest vomiting (moderate to severe symptoms) prevalence, i.e. 10 patients (8.3%), was reported to be at the second hour postoperatively.

As delineated in Table 4, vomiting frequency comparison between the intervention and control groups in various occasions (recovery time, 2, 4, 6, 8, and 24 hours postoperatively) showed that there was a lower frequency of vomiting in intervention group, but this difference was not statistically significant. 

## 5. Discussion

Our aim in this study was to assess the effects of short time fasting protocol on post adenotonsillectomy pain, nausea, vomiting, and instant oral feeding initiation in children hospitalized in Tabriz Children Hospital.

Adenotonsillectomy complications might increase the risk of aspiration which may complicate the patients (24). Numerous authors have searched for the ideal preanesthetic medication, and also for the best medication route. The premedication must be acceptable to patients, and an atraumatic route of administration should be available, in addition to the other characteristics required for such a drug (25, 26).

An effective pain therapy to block or modify the physiological responses to stress has become an essential component of modern pediatric anesthesia and surgical practice (27).

Comparing oral feeding initiation between the two groups revealed that there was an earlier oral feeding initiation among intervention group compared to the control group, i.e. patients who had received oral dextrose prior to the operation were able to start postoperative oral feeding earlier than those who were kept fasting as department routine preoperatively. Among control group, the number of patients who had started oral feeding in the first hour postoperatively was significantly lower than the intervention group. Also the number of patients starting oral feeding in the following occasions was higher in the control group.

Carithers et al. (28) and Guida et al. (29) believed that postoperative nausea is the most common postoperative complication in children despite advancements in surgery and anesthesia. Different surveys have reported prevalence rates ranging from 62% to 73% (30, 31).

In our study, the prevalence of these two complications, nausea and vomiting, in various postoperative hours was different. Highest nausea prevalence was reported to be at recovery time and two hours after the operation, which generally affected 21 patients (17.5%), a lower rate in comparison to the former studies, and the highest vomiting prevalence after adenotonsillectomy was at the second postoperative hour with a lower rate in comparison to the former studies. Hamid et al. (15) reported a vomiting prevalence rate in 80% for post adenotonsillectomy children who did not receive any prevention for vomiting.

In this survey, the frequency of Acetaminophen administration for intervention group was significantly lower than the control group, which could indicate the fact that among intervention group, patients’ pain degrees were milder than the control group, and they experienced a better recovery than the control group. As like as our findings, Nygren stated in his study that despite some former surveys which believed that preoperative fasting time limitation and hydration of patient do not affect his or her status postoperatively, but it seems to be effective in patient condition and recovery. This survey claimed that preoperative hydration of the patients is significantly effective in recovery, lowering nausea and vomiting, and earlier discharge of patient from hospital. This study also showed that a carbohydrate serum preparation prior to the operation is significantly effective in recovery after the operation (32). In another study Dr. Seyedhejazi and his colleagues (33) reported that, infiltration of bupivacaine and clonidine in children undergoing tonsillectomy is more efficacious than single IV fentanyl to decrease postoperative pain. This approach is also safer regarding the intraoperative complications.

Findings showed that in all postoperative occasions (recovery time, 2, 4, 6, 8, and 24 hours postoperatively) pain severity in the intervention group was less than the control group. Similar finding were reported by Klemetti et al. (34). In the mentioned study the intervention group that experienced shorter fasting time period than control group members experienced more severe pain (P = 0.0002) (34). None of the two groups differed in nausea degree in the post anesthesia care unit, but as time passed, nausea and vomiting level increased in the both groups. Although there was no statistically significant difference in nausea and vomiting between the two groups, but there was a higher frequency and severity in the control group.

This study stated that shorter preoperative fasting time plus suitable and controlled feeding in patients has a significant decreasing effect on postoperative pain, and this intervention could increase patients’, particularly children’s resistance to postoperative nausea and vomiting (34).

As in Klemetti’s survey (34), in our study there were significant differences in pain, but no significant difference in postoperative nausea and vomiting. Considering nausea severity in various postoperative occasions, there was a significantly lower nausea in intervention group, only in recovery time.

In recovery occasion, only four patients of the intervention group complained of nausea, compared to 17 in the control group. In other postoperative occasions (2, 4, 6, 8, and 24 hours postoperatively) nausea severity was lower in the intervention group than the control group, but this difference was not statistically significant. We think that our study limitation is its small study population, and also usage of cold knife techniques for adenotonsillectomy, so we suggest further multi central studies in large groups and with other scales for comparison of study groups and usage of different surgical instruments for adenotonsillectomy.

This study showed that shortening of preoperative fasting period and hydration of the patient few hours prior to the adenotonsillectomy may lower postoperative pain and accelerate oral feeding initiation time. Nevertheless this method lonely does not seem to be effective in prevention of postoperative nausea and vomiting.
