# Corrigendum to: Is Fasting Duration Important in Post Adenotonsillectomy Feeding Time? [Published in Anesth Pain Med. 2014 January; 4(1): e10256]

**DOI:** 10.5812/aapm.18021

**Published:** 2014-02-13

This corrigendum/addendum supplies corrected statements and proofs of an error by author.

Hereby, authors declare that the corrected name and affiliation of the fifth author is:

Samad EJ Golzari ^3^

^3^ Cardiovascular Research Center, Tabriz University of Medical Sciences, Tabriz, Iran

*Dr. Mahin Seyedhejazi*

*Corresponding author*

